# Challenges of primary health care leadership during the COVID-19 pandemic in Sweden: a qualitative study of managers’ experiences

**DOI:** 10.1108/LHS-08-2022-0089

**Published:** 2023-02-16

**Authors:** Janna Skagerström, Hanna Fernemark, Per Nilsen, Ida Seing, Maria Hårdstedt, Elin Karlsson, Kristina Schildmeijer

**Affiliations:** Regional Executive Office, Region Ostergotland, Linkoping, Sweden and Department of Health, Medicine and Caring Sciences, Linköping University, Linköping, Sweden; Department of Health, Medicine and Caring Sciences, Linköping University, Linköping, Sweden and Primary Health Care Center, Lambohov, Region Ostergotland, Linkoping, Sweden; Department of Health, Medicine and Caring Sciences, Linköping University, Linköping, Sweden; Department of Behavioural Sciences and Learning, Linköping University, Linköping, Sweden; Vansbro Primary Health Care Center, Region Dalarna, Falun, Sweden and Center for Clinical Research Dalarna, Uppsala University, Uppsala, Sweden; Department of Health, Medicine and Caring Sciences, Linköping University, Linköping, Sweden; Department of Health and Caring Sciences, Linnaeus University, Vaxjo, Sweden

**Keywords:** Health care, Leadership, Management, General practice, Crisis leadership

## Abstract

**Purpose:**

At the outbreak of the COVID-19 pandemic, health care was at the centre of the crisis. New demands made existing organizational practices and services obsolete. Primary health care had a great deal of responsibility for COVID-19-related care. The pandemic demanded effective leadership to manage the new difficulties. This paper aims to explore experiences and perceptions of managers in primary health care in relation to their efforts to manage the COVID-19 crisis in their everyday work.

**Design/methodology/approach:**

The authors used a qualitative approach based on 14 semi-structured interviews with managers in primary health care from four regions in Sweden. The interviews were conducted during September to December 2020. Data were analysed using conventional qualitative content analysis.

**Findings:**

Data analysis yielded three categories: lonely in decision-making; stretched to the limit; and proud to have coped. The participants felt lonely in their decision-making, and they were stretched to the limit of their own and the organization’s capacity. The psychosocial working conditions in primary care worsened considerably during the pandemic because demands on leaders increased while their ability to control the work situation decreased. However, they also expressed pride that they and their employees had managed the situation by being flexible and having a common focus.

**Originality/value:**

Looking ahead and using lessons learnt, and apart from making wise decisions under pressure, an important implication for primary health-care leaders is to not underestimate the power of acknowledging the virtues of humanity and justice during a crisis. Continuing professional education for leaders focusing on crisis leadership could help prepare leaders for future crises.

## Introduction

Coronavirus disease (COVID-19) has had an unprecedented global impact on societies worldwide. At the outbreak of the pandemic, health care was at the centre of the crisis. Challenging new demands made existing organizational practices and services obsolete. In the 21 health-care regions in Sweden, major adjustments to traditional ways of organizing and providing health care were needed [Rawaf *et al.*, 2020; Swedish Association of Local Authorities and Regions (SALAR), 2020a]. Based on the decentralized system of health care, the regions decided what adaptations to make and how they would provide expanded pandemic-related patient services with existing resources [Swedish Association of Local Authorities and Regions (SALAR), 2020b].

Primary health care had to shoulder a great deal of responsibility for COVID-19-related care (Huston *et al.*, 2020; World Health Organization, 2021). Among these tasks were reinforcement of the general public health messages, diagnostics, treatment and advice to an increasing number of patients with symptoms of respiratory infection and organization of COVID-19 testing and vaccination. Primary health care also became important in helping patients to cope with anxiety concerning the virus and reducing the demands on hospital services (Dobson *et al.*, 2021; Chew *et al.*, 2020; National Board of Health and Welfare, 2021). Many patients avoided seeking primary health care due to the pandemic, which resulted in unpredictable patient flows, postponement of regular care and the risk of increased morbidity (National Board of Health and Welfare, 2020). The challenges facing primary health care were unpredictable for staff and leaders (O’Mahony, 2020).

The pandemic required effective leadership in primary health care to manage the difficulties created by the pandemic. Leadership can be defined as a social process occurring in a group whereby an individual demonstrates the ability to guide a group towards achieving a common goal (Yukl, 2013). It is widely recognized that adequate leadership is a necessary requirement in the health- care sector. For example, effective leadership has been associated with improved quality of care, clinical outcomes, integrated care delivery, healthy work-practice settings for clinicians and improved retention of staff (Reichenpfader *et al.*, 2015). The pandemic highlighted the importance of crisis leadership to organize and provide primary health care without endangering the safety or quality of the care provided. Crisis leadership requires different skills than day-to-day work because the leaders need to be well prepared to manage unknown situations (Wilson and Newstead, 2022).

When the pandemic hit Sweden in March 2020, staff and leaders in primary health care and other health-care settings found themselves in uncharted territory. It is important to document and report on leaders’ experiences concerning the challenges in organizing and providing care during the pandemic. We have not been able to identify any empirical studies on the issue of leadership in primary health care during the pandemic. Therefore, the aim of this study was to explore the experiences and perceptions of managers in primary health-care centres in relation to their efforts to manage the COVID-19 crisis in their everyday work. The findings may be used to inform continuing professional education for managers and prepare leaders for future health crises and other unpredictable situations. The study may also contribute with insights into how difficult priorities can be made and how health-care systems might be modified under pressure from scarcity of resources and increasing demand for services.

## Methods

### Study design and setting

We used a qualitative approach based on semi-structured interviews with managers in primary health care, defined as employees who had a management role (Swedish Code of Statutes, 2017, p. 30), to gain a deeper understanding of the leaders’ experiences and perceptions of work and the work situations during the pandemic. The interviewees were used at the local level of a primary health-care organization/primary health-care centre responsible for basic medical care, prevention and rehabilitation not requiring the medical and technical resources of hospitals or other specific skills (National Board of Health and Welfare, 2016).

Leadership is usually defined in terms of a process of influence to achieve certain goals. The concept is not to be confused with management; leadership can be considered a social process or a relationship, while management is a position (Yukl, 2013). However, in a Swedish context, managers in primary health care can be considered to be leaders since they set the direction for primary health care and directs and distributes the work.

The Swedish health-care system is divided into 21 geographic and organizational regions, providing health care for a total population of about ten million people in Sweden. The health-care system is funded primarily by public means and residents are insured by the state and have equal access to health care. Out-of-pocket fees are low and regulated by law. Private health care also exists. In 2019, there were 1,140 primary health-care centres in Sweden (SALAR, 2020b).

### Recruitment of participants

A purposeful sampling strategy was used to achieve a diverse sample of primary health-care centres with regard to size (number of listed patients) and location (rural, urban and different regions of Sweden). Six regions were approached by email to recruit participants. Four regions agreed to participate. In two regions, participants received information about the study through the primary health-care management. In another region, four primary health-care centres were chosen based on our sampling strategy by the primary health-care management. In the fourth region, we established contact with a researcher/clinician who disseminated information about the study to relevant primary health-care centres. We approached 15 leaders, of which 14 agreed to participate.

### Data collection

The authors developed a semi-structured interview guide, which included questions about changes linked to the pandemic and what had affected them the most. There were also questions about how the changes had affected their own work environment, how they experienced that relationships in the workplace had changed and what we could learn from the pandemic. The questions include, for example: What changes have been made at your workplace with regard to the coronavirus pandemic? What changes have affected you the most? Can you describe how the changes have affected your working conditions? Was the psychosocial relations at your primary care centre affected by the pandemic – if so, how? Was cooperation with other units affected? Exploratory questions were also used to deepen our understanding of the respondents’ answers.

We conducted the interviews via Zoom during September to December 2020. This was a period of many workplace restrictions due to the pandemic, and on-site interviews were not possible. The interviews lasted between 28 and 55 min and conducted by XX and XX. All interviews were digitally recorded and transcribed verbatim by a professional firm specialized in transcribing interviews. The transcripts were examined for accuracy by the interviewers.

### Data analysis

Data were analysed using conventional qualitative content analysis as described by Hsieh and Shannon (2005). In conventional content analysis, categories are based on participants’ experiences and are derived from the data. In the first step, all interviews were read by three of the authors (XX, XX and XX) and the rest of the authors each read at least one-third of the interviews. This was done to get a sense of the whole. In the second step, exact words from the text that captured information important to answer the aim of the study were highlighted, and researchers XX and XX wrote down their overall impressions of the material. All authors discussed and compared the impressions. Thereafter, XX and XX continued the process and coded the highlighted text to create an initial coding scheme. When the codes had been sorted into subcategories and categories, these were presented to all the authors. Some changes were made to the content of a few subcategories after discussion. In the final step, categories and subcategories were described, and quotes were selected to illustrate each subcategory.

### Ethical considerations

The study was approved by the Swedish Ethical Review Authority (no. xxxx-xxxxx) and registered in line with the General Data Protection Regulation in Sweden. Information regarding the aim of the study, various practical and ethical issues concerning the interviews (including data storage) and that it was possible to withdraw at any point during the interview was sent to the participants. The participants provided informed consent, confirming that they had read and understood the information they were given.

## Results

Fourteen managers working in primary health care participated in the study. The managers were employed in units in both urban and rural areas. All of them had a background in health care; they included eight nurses, three physicians and three belonging to other occupational categories (Table 1). The majority of the managers did not have any clinical role in their current position. However, three of the managers expressed that they worked clinically on rare occasions or when there was a need to step in due to heavy workload at the centre.

Data analysis yielded three categories consisting of nine subcategories (Table 2).

### Lonely in decision-making

The category “lonely in decision-making” concerns the managers’ experience of feeling loneliness in implementing and prioritizing the changes and adjustments that the new pandemic situation required. Although a considerable number of directions and recommendations came from the central health-care management, the managers experienced that they had to solve most of the practical issues on their own. A lack of collegial support in decision-making and practical matters, from other managers or the superior management, were described. At the same time, they had to be supportive to their staff, which required skills to identify and frame the situation and to help employees understand the manager’s priorities.

### Social support

Some participants felt they lacked support, which they perceived as an additional burden, but others believed that they received sufficient support from their region, which has central responsibility for primary health care and their work/care. Managers occasionally felt isolated from management colleagues who had previously provided support and opportunities to discuss difficult issues:

I’m also angry, because I don’t think I’m getting support, I don’t think I’m getting help […] from superior managers. My manager has over 20 managers under her […] she doesn't have a chance […] and she never has time to talk to me, or […] so I'm quite lonely (ET8).

Providing support to their employees was an important but time-consuming task for the managers during the pandemic. Managers stressed the importance of remaining calm and collected in front of the employees and taking the time to talk to everyone. Some managers had mixed feelings when they had to remind colleagues of restrictions and regulations despite wanting to be supportive and physically close to them.

### Managing changes

The participants openly shared their experiences of the organizational changes that took place during the pandemic. Many changes appeared abruptly due to new routines and guidelines from above. The managers expressed that they had to solve problems instantly and had to revise work routines from one day to the next to keep up with new directions. Each unit had to solve practical issues regarding facilities and staff to adhere to the directions from superior management.

Organizational changes and new routines influenced cooperation with other parts of the health-care organization. For example, primary care handled patients that would usually be treated in hospitals. In addition, cooperation with community-based services became more intense with meetings regarding how to reduce the spread of virus and conduct test in home care and nursing homes, etc. Simultaneously, social distancing regulations meant that forums, where managers used to meet, became digital. Some managers expressed that this reduced collegial support as well as cooperation:

I think that there is a very interesting aspect here about what leadership becomes if you cut off all contact […] with the outside world, that it is more difficult to lead, and I don’t think you will see […] the effects of that until maybe three years from now […] in relationships, that is collaborative relationships., because you haven't had the opportunity to build then. And let’s hope that the pandemic doesn’t […] keep us stuck that long (JP42).

Digital solutions provided opportunities to interact with patients without having physical contact. Managers described that it was sometimes stressful to set up digital health-care meetings at short notice and argued that this option was not suitable for all patients. However, for others, the shift to more digital patient consultations made it easier to carry out their work tasks:

[…] adults who are quite IT savvy can function, but we can't call up patients who are not so […] high tech. The region has a bit of a problem with creating systems that are simple […] one might think […]. and it […] is not possible for health care staff to be technicians and support patients […] in technology, before they can even start their visit (HM14).

### Ad hoc decision-making

The experience of being a sole decision maker varied among the managers. Although most of them experienced the situation as burdensome, there were also statements about growing with the task and a sense of freedom to be the one who actually made the final decisions and had influence. Having to make decisions that could affect patient safety or safety for the employees without having sufficient knowledge about the virus or the disease at hand was stressful. Furthermore, many decisions had to be made quickly or revised as new information emerged:

I think maybe the emotional pressure is the biggest [stressor] […] dealing with difficult, quick decisions and constantly feeling like “Help, what happens tomorrow? Do we have any staff in place at all? How are we going to manage all phone calls? What can we redirect?”’ (ET8)

The decisions on how to adapt new routines to the circumstances at each primary health-care centre often had to be made quickly. The managers expressed a feeling of having decisional latitude in making independent decisions, which some managers considered advantageous. However, the rapid changes also created challenges. Sometimes patients heard about new regulations or restrictions through the media and expected them to be implemented immediately in primary health care:

And then when the politicians have said, well […] have said something in the media or […] that the politicians have decided something […] then it doesn’t give us an opportunity to have a […] perspective before then must be delivered. And politics and care […] or politics and patient safety, it’s not the same thing […] it’s not the same thing at all, there’s no […] thought there […] or evidence base, or anything, but it […] it feels like, it’s too far apart. (FH13)

### Stretched to the limit

The participants felt that they were approaching the limit of their own and the health-care centre’s capacity. They believed that many situations had to be resolved from day to day at the expense of the long-term perspective of the work at their unit. This way of working might be manageable in the short term, but they thought it would be difficult to sustain it in the longer term.

### Intense information flow

The vast amounts of information every day were perceived as almost unmanageable, causing feelings of exhaustion. Furthermore, the information came from many different sources, which made managers feel afraid to miss vital information or spread information that was unimportant or already outdated. To reduce the flow of information passed on to the employees, some of the managers screened and excluded parts of the information that they did not consider necessary before distributing the information:

I can say that that’s been the difficulty […] really, that there have been so many different directives […] and it’s difficult to keep up to date. Where I work, my manager might send it, then the chief surgeon sends it, then the chief physician sends it, and then in the end you don't know, ‘Hello! Have I read this, or not?’ You have so much to read, so much to see […] which was first and they were maybe dated at the same time […] so that it was very, there were very many directives. (MB24)

### Staff well-being

The health consequences of the COVID-19 pandemic affected not only patients but also health-care professionals at the primary care units. Sick leave among the employees in the workplace was high, and the managers had to spend considerable time and effort trying to find replacements for those who were unable to work. Restrictions that did not allow people to meet and socialize in the same way as before affected the atmosphere and relationships in the workplace. Social activities with colleagues were cancelled, and the high workload meant fewer opportunities to debrief and talk with each other, which was detrimental to the work climate.

Some managers also reported that employees kept an eye on how others were complying with guidelines and got annoyed if someone broke restrictions, even if it was during their free time:

You got annoyed by what people did on weekends […] if they went to Stockholm […] your private life became more involved at work […] how you behaved, and as a manager you got […] a lot of criticism […] what I did with my children and […] what I did in my private life. It was very important for me to stick exactly to the Public Health Authority, no more and no less. I couldn't do more […] because I had to really stand up for what was said [by the authorities], even in my private life […] and show that I did that. (FH13)

### Short-term focus

Prioritizing was considered as an everyday manager task. During the pandemic, however, it emerged that the managers made different priorities than they usually did. Projects and development work that could be beneficial for the patients in the future had to be postponed. Strategic work and long-term plans were put aside in favour of day-to-day management. Further, annual check-ups for some patients with chronic diseases had to be postponed during the pandemic, which was not always well received by the patients:

I think it has gone well, but it has also required us to put a lot of other things aside […] and not just me personally but development work […] and […] other things that perhaps primary care would have liked to do during this year. (JP42)

### Workload

The managers themselves had a lot of work to do keeping up with and distributing information, planning schedules and new routines, etc. There was no time for reflection, and the managers expressed that they continuously felt they were one step behind. Some of the informants reported working long hours and having a hard time letting go of work when they were off duty. Others felt they could unwind when they left work and that they had time to recover during weekends. The fear of becoming infected and having to isolate oneself led to an increased awareness of symptoms, which could also contribute to stress:

I’m worried about the organization and it […] it’s eating at me […] I’m worried, how long will people last? We haven’t had a holiday this summer, many of us. We had to cancel holidays and go to work. We […] work both in the evenings and at weekends sometimes. I’m worried about what will happen to our tired staff […] it’ll be a damn long winter. And then constantly working understaffed, because that’s what we do all the time, because there are always some sick, and some who are at home […] that, I’m really worried about that. (ET8)

### Proud to have coped

The participants expressed a considerable sense of pride in themselves and their staff at being able to cope with the new situation. Despite the difficult circumstances, they had managed to continue to work based on guidelines and find new solutions, both in terms of their own primary health care unit but also regarding collaboration with others.

### Flexibility

The managers expressed that themselves, employees and health-care providers working within other organizations had been more flexible than they had expected. Some of the employees had shown unexpected adaptability when they stepped forward and offered to perform more than their normal tasks. The employees also adjusted well and quickly to guidelines and routines and helped to solve upcoming situations:

[…] and then it has been withdrawn after only a few days, it has […] has been incomplete routines you could say. It’s moved very quickly, so it’s been almost a strategy of ‘Let’s sit on the shelf and wait a couple of days and see what happens. We’ll have time to […] change our […] routines, because how we should do things will change.’ Yes, but then I’ve had […] well, the staff have changed everything very quickly, they’ve been fantastic at changing things, at […] being on the ball, at learning the ropes, so that part has worked very smoothly, they’ve been […] on their toes and […] have learned to adapt as well […]. (HH40)

Some of the managers expressed that cooperation with units outside their own primary health-care centre functioned well and improved. For example, several primary health-care centres worked together, and there was more cooperation with municipal health care.

During the pandemic, digitization was accelerated at a pace that the managers did not think would have been possible under normal circumstances. Both health-care professionals and patients saw a need and the advantages of new solutions and were mostly positive to these changes. However, some of the managers experienced resistance from certain groups of personnel about the introduction of more digital care meetings, for example:

Everyone understood that we needed to go digital, there might have been more resistance if we had done it during a quiet period when we didn’t have COVID-19. So it was certainly helpful that […] everyone understood, the general population as well […] it probably helped that we were on the same page all of us. (FH13)

### Shared focus

It emerged in the interviews that the managers experienced that many work tasks were easier to solve compared with before the pandemic. The reason for this was that during the pandemic, both the employees not only in their own unit but also in other health-care units had gained a shared mission. Conflicts were put aside to jointly cope with the new situation and all the tasks and assignments that were now required of all of them:

And […] I thought it was visible on many levels. It was a bit like that, and also […] internally in our building, that […] you stop […] yes you focus on common solutions of the challenges that exist. You put some things aside for a bit, because they don’t have priority right now; so it must be, and you in some way mutually agree that it doesn’t. And in that way, I think that many conflicts have been lifted […] that it has become a better climate. (ASK21)

Cooperation outside the regular limits increased when everyone was working towards the same goal and focused on solving the same problems. People worked together across professional and organizational boundaries and were more inclined to think outside the box to find new solutions, contributing to an increased feeling of belonging. Further, the common focus on certain issues, such as hygiene and digital meetings, made it possible to reach out and implement new routines and tools much more quickly than would have been the case otherwise, according to the managers:

You’ve seen the staff, how good they are at adjusting and rethinking […] you can really see who thinks, what can I say, operationally and solves problems […] you see who is […] well […] and this is a boring word […] flexible. (PS5)

## Discussion

This study aimed to explore experiences and perceptions of primary health-care leaders in Sweden in relation to their efforts to manage the COVID-19 crisis. The participants felt lonely in their decision-making and stretched to the limit of their own and their organization’s capacity. However, they also expressed pride that they had coped with the new situation. Managers highlighted that prioritizing was an everyday challenge, including an intense information flow that was perceived as almost unmanageable. The fact that the workload was also high due to changed routines, new tasks and high sick leave rates among the staff, further increased the pressure, according to the participants.

The findings of our study are broadly consistent with earlier research on leadership in crises. It is well established that effective leadership during crises is crucial, but it can often be complex due to the unpredictability of such situations (Muffet-Willett and Kruse, 2009). This unpredictability was evident in the participants’ experiences of information changing from day-to-day, lack of knowledge about the virus and uncertainty regarding the staff situation including sickness absence.

Crisis leadership research has shown the need for integrated models of leadership in crisis situations (Boin *et al.*, 2013). Crisis leadership involves the ability to oversee planning and execute tasks and processes in response to crises. Leaders in a crisis must make wise and timely decisions, despite conditions of ambiguity and pressure (Wilson and Newstead, 2022). However, crisis leadership also entails adaptive capabilities to address the human aspects of crisis, e.g. needs, emotions and behaviours of people taking action to address, prevent, mitigate and recover from crises. An emphasis on the virtues of humanity and justice, such as issues of caring, empathy, mutual responsibility and a sense of unity, could facilitate management during a crisis (Wilson and Newstead, 2022). Both planning capabilities (and lack thereof) and capabilities concerning human aspects were evident in our study as the participants expressed that they prioritized managing the pandemic situation, including being supportive of the health-care staff and thereby down prioritizing other tasks such as development work.

Research on leadership during crises has also shown the importance of communication and collaboration in crisis situations (Baker *et al.*, 2020; Kakemam *et al.*, 2020; Kapucu and Ustun, 2017; Kaul *et al.*, 2020). For instance, by acknowledging employees’ needs, their distress was alleviated and trust in the leaders was enhanced, which, in turn, enhanced employees’ acceptability of the leaders’ decisions. Encouraging humanity and justice in the organization also enhanced the collective capability among the employees to face the mutual challenges posed by the crisis, empowering employees because they all had a useful role to play and were in it together (Wilson and Newstead, 2022). These findings are in line with the results of our study whereby managers experienced employees taking steps forward with increased responsibilities to be able to manage the situation. The participants saw employees develop and grow with the task. The participants in the present study also highlighted how the focus on managing the pandemic tended to make them and their employees put conflicts aside, and that creative solutions and cooperation with other units increased. However, the participants lacked collegial support from other managers and felt lonely in their decision-making. Potentially an even more crisis-oriented leadership, including more collaboration and support between managers at different levels, could have facilitated decision-making and the sense of support among managers in primary health care during the pandemic. The managers could have benefitted from help with filtering the extensive amount of information. In a crisis, leaders should articulate in practical terms what actions are needed, and how they serve the collective good in the organization (Wilson and Newstead, 2022). However, looking ahead and using the lessons learnt, an important implication for primary health-care leaders, apart from making wise decisions under pressure, is not to underestimate the power of acknowledging the virtues of humanity and justice during a crisis.

The findings of our study suggest that the psychosocial working conditions in primary care worsened considerably during the pandemic because the demands on the leaders increased while their ability to control the work situation decreased. The widely used job demand control model posits that jobs that are high on demands and low on control, i.e. “high-strain jobs”, carry a high risk of adverse psychological symptoms such as anxiety and depression (Karasek and Theorell, 1990). The model was later extended by Johnson and Hall (1988), who added the dimension of support at the workplace, which has a “buffering” effect by moderating the negative impact of high strain. The leaders experienced loneliness in their decision-making, suggesting that their work situation was characterized by high demands and low social support, which is considered the most harmful combination for health and well-being (Johnson and Hall, 1988). The findings point to an imbalance between the leaders’ efforts and the rewards they received during the pandemic, which implies an increased risk for stress. The effort-reward imbalance model postulates that individuals invest efforts and expect rewards in return, and failed reciprocity resulting from a violation of this norm of return expectancy increases the risk for poor health and well-being (Siegrist, 1996). The leaders’ loneliness in decision-making and stretched capacity increased the effort they had to put into their work, but the pride they expressed in being able to cope with the new situation may have been an important reward that reduced the imbalance somewhat.

The study has some limitations that should be considered when interpreting the findings. The managers participating in the study showed interest in being interviewed and volunteered to do so. This might have affected the results because managers who did not volunteer might have experienced even more stress or lack of time than the participants. Further, it is possible that the participants had more positive experiences from leadership during the pandemic that they wanted to share. Regardless, the results might be of interest because they reflect the thoughts of several managers working in different regions in Sweden, in both rural and urban areas, with different professional background and diverse ages. Another limitation is that the interview guide used was not designed exclusively for interviews with managers; the same guide was used to interview health-care professionals working clinically in primary health care. Designing an interview guide exclusively for the managers might have given even more information pertaining to leadership and management concerns. However, the managers described their own work situations in detail and the authors performing the interviews were prompted with questions to get more information. The power of information is affected by the study aim, sample specificity, use of an established theory or framework, quality of the dialogue in the interviews and the analysis strategy (Malterud, 2016). The interview data provided rich information. Further, the credibility of the results was enhanced by the multidisciplinary research team representing several professions and research areas.

In conclusion, using lessons learnt, an important implication for primary health-care leaders, apart from making wise decisions under pressure, is to encourage the employees’ ability to grow with the task and to adjust towards common goals during a crisis. Continuing professional education for leaders focusing on crisis leadership could help prepare leaders for future crises.

## Figures and Tables

**Table 1.** Characteristics of the participants

Characteristic	No. (%)
*Sex*	
Female	13 (93)
Male	1 (7)
*Profession*	
Physician	3 (21,4)
RN	8 (57)
Other	3 (21,4)
*Age*	
30–39 years	1 (7)
40–49 years	6 (43)
50–59 years	5 (36)
60–69 years	2 (14)
*Location of workplace*	
Urban	6 (43)
Rural	8 (57)

**Table 2.** Categories and subcategories

Lonely in decision-making	Stretched to the limit	Proud to have coped
Social support	Intense information flow	Flexibility
Managing changes	Staff well-being	Shared focus
*Ad hoc* decision-making	Short-term focus	
	Workload	
